# Flufenamic Acid Inhibits Adipogenic Differentiation of Mesenchymal Stem Cells by Antagonizing the PI3K/AKT Signaling Pathway

**DOI:** 10.1155/2020/1540905

**Published:** 2020-03-16

**Authors:** Xuenan Liu, Zheng Li, Hao Liu, Yuan Zhu, Dandan Xia, Siyi Wang, Ranli Gu, Ping Zhang, Yunsong Liu, Yongsheng Zhou

**Affiliations:** ^1^Department of Prosthodontics, Peking University School and Hospital of Stomatology, National Laboratory for Digital and Material Technology of Stomatology, Beijing Key Laboratory of Digital Stomatology, National Clinical Research Center for Oral Diseases, 22 Zhongguancun South Avenue, Beijing 100081, China; ^2^Department of Materials Science and Engineering, College of Engineering, Peking University, Beijing 100871, China

## Abstract

**Objectives:**

Flufenamic acid (FFA) is a representative of the fenamic acids, an important group of NSAIDs. In the present study, we study the effects of FFA on adipogenesis of human mesenchymal stem cells (MSCs) and we explore the potential mechanism.

**Methods:**

To investigate the effects of FFA on adipogenic differentiation of hMSCs, human adipose-derived stem cells (hASCs) and human bone marrow mesenchymal stem cells (hBMMSCs), representative of hMSCs, were treated with FFA during adipogenic differentiation *in vitro*. The effects of FFA *in vivo* were evaluated using a heterotopic adipose formation assay in nude mice as well as ovariectomized (OVX) and aged mice. To explore the mechanism of FFA, Western blot was used to determine activation of the PI3K/AKT signaling pathway.

**Results:**

Our results demonstrate that, at certain concentrations, FFA inhibited adipogenesis of human MSCs both *in vitro* and *in vivo*. Mechanistically, FFA inhibited adipogenesis of human MSCs by inhibiting the PI3K/AKT pathway.

**Conclusions:**

The present study indicated that FFA could be used to inhibit adipogenesis of human MSCs in tissue engineering and diseases related to excessive adipogenic differentiation of MSCs.

## 1. Introduction

Tissue engineering has become a hotspot in recent years [1]. As a commonly used seed cell in tissue engineering, mesenchymal stem cells (MSCs) which have multipotent differentiation potential play an important role [2–6]. Therefore, much focus has been placed on how to regulate the lineage commitment of MSCs. Adipogenic differentiation is one of the differentiation direction of MSCs which has important physiological and pathological significance [2, 3]. Excessive adipogenic differentiation of MSCs can be seen in several diseases, such as obesity [7, 8] and osteoporosis [9]. Thus, how to regulate the adipogenesis of MSCs has attracted more and more attention.

Nonsteroidal anti-inflammatory drugs (NSAIDs) are widely used for anti-inflammatory and analgesic purposes. NSAIDs inhibit cyclooxygenases (COXs) which inhibit the synthesis of prostaglandin [10–12]. NSAIDs are a large group of molecules, and the chemical structures of the different members vary widely, leading to their diverse biological effects in addition to anti-inflammatory and analgesic properties. NSAIDs can be divided into several groups according to their chemical structure; examples of these are anilines, acetic acids, salicylic acids, and fenamic acids [13]. Several NSAIDs have complicated effects on proliferation, adhesion, spreading, migration, and other biological behaviors of MSCs, including aspirin [14] indomethacin [15, 16], and ibuprofen. Among them, indomethacin promotes adipogenesis of MSCs [16]. However, little is known about the effects of the fenamic acids on adipogenic differentiation of hMSCs.

The lineage commitment of MSCs is regulated by a variety of transcription and signaling factors, including the AKT signaling pathway. AKT signaling plays an important role in cellular proliferation, differentiation, and apoptosis [17], as well as in tumorigenesis [18]. The AKT pathway is upregulated during adipogenic differentiation [19–21]. Furthermore, impaired adipogenesis is seen in Akt1/Akt2 knockout mice, and both Akt1 and Akt2 are necessary to induce PPAR*γ* [22], which is a key regulator of adipogenesis. Whether fenamic acids can regulate the adipogenesis of MSCs via the AKT pathway remains unclear.

In the present study, we investigate the *in vitro* and *in vivo* effects of flufenamic acid (FFA), a representative of the fenamic acids, on the adipogenesis of human MSCs. We report a potential mechanism of action, which provides valuable insight into the potential use of FFA in tissue engineering and diseases related to excessive adipogenic differentiation of MSCs.

## 2. Methods

### 2.1. Culture and Adipogenic Induction of hASCs and hBMMSCs

Primary hASCs and hBMMSCs were obtained from ScienCell Research Laboratories (San Diego, CA, USA). All cell-based *in vitro* studies were repeated at least three times, using hMSCs from three different donors.

FBS, DMEM, *α*-MEM, and 100x penicillin and streptomycin mixture were obtained from Gibco (Grand Island, NY, USA). Human ASCs and BMMSCs were cultured at 37°C with a 5% CO_2_ atmosphere in proliferation medium (PM), consisting of penicillin/streptomycin, 10% (*v*/*v*) FBS, and either fresh DMEM (for hASCs) or *α*-MEM (for hBMMSCs). The adipogenic medium (AM) was comprised of DMEM or *α*-MEM containing 10 *μ*M insulin, 100 nM dexamethasone, 200 *μ*M indomecin, 500 *μ*M 3-isobutyl-1-methylxanthine (IBMX), 10% (*v*/*v*) FBS, and penicillin/streptomycin.

### 2.2. Preparation of Concentrated FFA Solution

In order to examine the effects of FFA at different concentrations on the adipogenesis of hMSCs and to identify the optimal concentration for adipogenesis of hMSCs *in vitro*, we dissolved flufenamic acid in DMSO to obtain a concentrated FFA solution of 200 mM FFA.

### 2.3. Oil Red O Staining

Cells were inoculated and cultured in PM and AM separately, and Oil red O staining was performed on the 21st day after adipogenic induction. Briefly, cells were fixed with 10% formalin for 1 hour and rinsed with 60% isopropanol, and 0.3% Oil red O working solution was applied. The cells were then washed with distilled water, observed, and photographed under a microscope. For quantitative assessment, stained cells were eluted with 100% isopropyl alcohol and quantified by spectrophotometric absorbance at 520 nm against a blank (100% isopropyl alcohol).

### 2.4. Real-Time Reverse Transcriptase-Polymerase Chain Reaction

TRIzol reagent (Invitrogen, Carlsbad, CA, USA) was used to extract total RNA from hASCs and hBMMSCs cultured *in vitro*. Afterwards, cDNA was reverse-transcribed using a PrimeScript RT Reagent Kit (Takara, Tokyo, Japan). Real-time quantitative PCR assays were conducted with SYBR Green Master Mix (Roche Applied Science, Mannheim, Germany) on an ABI Prism 7500 real-time PCR System (Applied Biosystems, Foster City, CA, USA). Glyceraldehyde-3-phosphate dehydrogenase (*GAPDH*) was used as a reference gene. The primer sequences used are described in Table 1.

### 2.5. Western Blotting Analysis

Human ASCs were washed with PBS and lysed in radioimmunoprecipitation assay (RIPA) lysis buffer containing 1% phosphatase inhibitor (Roche) and 2% protease inhibitor cocktail (Roche) at 4°C for 30 min. Cell lysates were sonicated and centrifuged at 13362g at 4°C for 30 min to obtain the supernatant. Total protein concentrations were determined using a Pierce BCA protein assay kit (Thermo Scientific). Aliquots (35 *μ*g) of total protein extracts were subjected to 10% SES-PAGE and transferred to a polyvinylidene fluoride membrane (Millipore, Billerica, MA, USA). Membranes were blocked in 5% skim milk for 1 hour and then incubated with anti-PPAR*γ* (Abcam, Cambridge, UK), anti-p-PI3K, anti-PI3K, anti-p-AKT, anti-AKT, or anti-GAPDH (Cell Signaling Technology, Beverly, MA, USA) overnight at 4°C. Subsequently, membranes were incubated with peroxidase-conjugated secondary antibody solution at room temperature. The immunoreactive protein bands were detected using an ECL kit (CWBIO, Beijing, China). Image J software (National Institutes of Health, USA) was used for quantitative densitometric analysis of protein bands. All protein levels were quantified by the target gene/GAPDH densitometric ratio.

### 2.6. *In Vivo* Implantation of hMSCs and Heterotopic Adipose Tissue Formation

This study was approved by the Institutional Animal Care and Use Committee of the Peking University Health Science Center (LA2016305). All experiments were performed under the approved guidelines.

All the mice were obtained from Vital River Corporation (Beijing, China). Mice were maintained in a pathogen-free facility on a 12-hour light/dark cycle with water and food provided *ad libitum*.

6-week-old (*n* = 24) female BALB/C homozygous nude mice were used for heterotopic adipose tissue formation. The 24 mice were randomly divided into four groups (6 mice per group): AM, AM+FFA of hASCs, and AM, AM+FFA of hBMMSCs. Human ASCs or BMMSCs were cultured in AM or AM with 25 *μ*M FFA for 1 week prior to inoculation in nude mice. The cells were then collected and incubated with a Collagen Sponge (Wuxi Biot Bioengineering Institute, Wuxi, China) (8 mm × 8 mm × 2 mm) at 37°C for 2 h. After centrifugation, the complex was implanted into the dorsal subcutaneous space of the nude mice. The implants were collected after six weeks and fixed with 4% paraformaldehyde. After that, each sample was cut in half for H&E staining and Oil red O staining.

### 2.7. Ovariectomy and Sham Operations

8-week-old (*n* = 40) female C57BL6 mice underwent ovariectomy (OVX) or sham operation. The forty mice were randomly divided into two groups (20 mice per group): Sham and OVX. Pentobarbital sodium (50 mg·kg^−1^) was used for general anesthesia by intraperitoneal injection. OVX or sham operations were performed on each mouse following standard procedure [23].

### 2.8. *In Vivo* Experiments with FFA Injection

After the operation, the 20 sham mice were randomly divided into two groups (10 mice per group): (1) Sham mice with normal saline (N.S.) and (2) Sham mice with FFA. Similarly, the 20 OVX mice were randomly divided into two groups (10 mice per group): (3) OVX mice with N.S. and (4) OVX with FFA. Four weeks later [24], FFA or N.S. was given to the mice by intraperitoneal injection once a day.

For aged mice, 12-month-old (*n* = 20) female C57BL6 mice were randomly divided into two groups (10 mice per group). Mice in group one (FFA) were given FFA once a day; mice in group two (N.S.) were given N.S., in an equivalent volume as the FFA solution, once a day.

Calculation of the *in vivo* treatment dose was established in our previous study [25].

One month after injection, the animals were humanely euthanized, and femur bones were collected, fixed in 10% formalin, and analyzed by H&E staining. 0.5 to 1 mm distal to the proximal epiphysis in the trabecular region of the femurs were chosen to analyse the parameters of adipocyte according to guidelines set by the American Society for Bone and Mineral Research (ASBMR) [26]. Adipocyte numbers and area per tissue area were analyzed on H&E staining images using SPOT5.3 Microscopy Imaging Software [27].

### 2.9. Statistical Analysis

Data are given as mean ± standard error (SE). SPSS Statistics 20.0 software (IBM) was used for statistical analyses. Independent two-tailed Student's *t*-tests, one-way ANOVA, and a Tukey's post hoc test were used to determine the level of significance. *P* values of *P* < 0.05 were considered statistically significant.

## 3. Results

### 3.1. FFA Inhibits Adipogenic Differentiation of hASCs *In Vitro*

**Figure 1:**
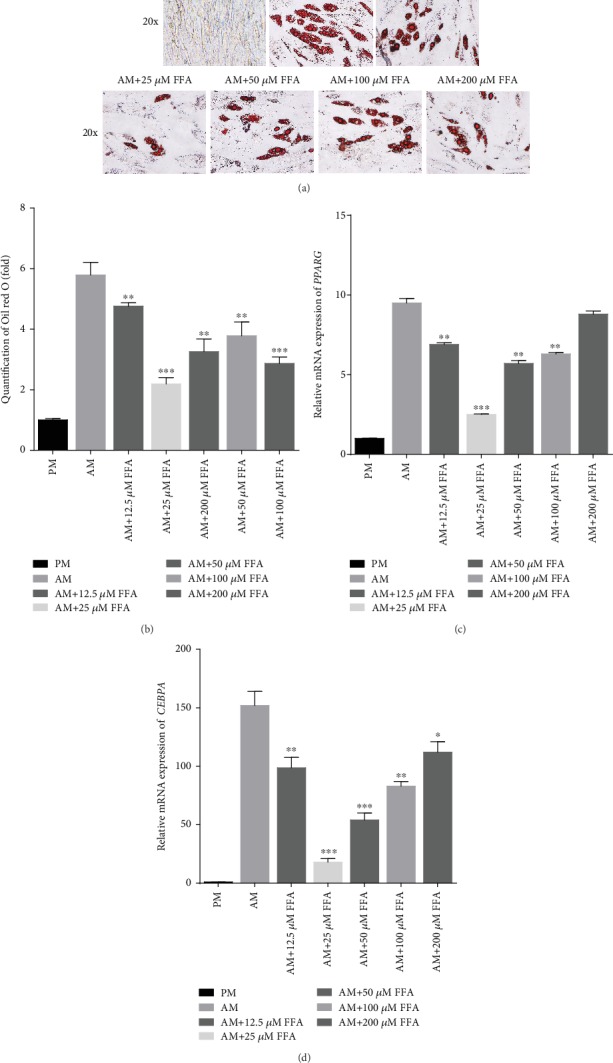
FFA inhibits adipogenic differentiation of hASCs *in vitro.* (a, b) Oil red O staining (a) and quantification (b) indicated that FFA at 12.5, 25, 50, 100, and 200 *μ*M inhibited lipid droplet formation in hASCs. Human ASCs were treated with PM, AM, or AM+FFA at 12.5, 25, 50, 100, or 200 *μ*M for 21 days before Oil red O staining and quantification. Scale bar = 100 *μ*m. (c, d) FFA at 12.5, 25, 50, 100, and 200 *μ*M inhibited the expression of *PPARG* (c) and *CEBPA* (d) in hASCs, as determined by qRT-PCR. All data are shown as the mean ± SE, *n* = 3. ^∗^*p* < 0.05, ^∗∗^*p* < 0.01, and ^∗∗∗^*p* < 0.001 compared with AM. PM: proliferation media; AM: adipogenic media; FFA: flufenamic acid; hASC: human adipose-derived stem cell; *PPARG*: peroxisome proliferator activated receptor gamma; *CEBPA*: CCAAT enhancer binding protein alpha; qRT-PCR: quantitative real-time reverse transcription PCR.

Oil red O staining and quantification indicated that 12.5, 25, 50, 100, and 200 *μ*M FFA had an inhibitory effect on the adipogenic differentiation of hASCs cultured in AM on day 21 (Figures 1(a) and 1(b)). Meanwhile, qRT-PCR showed that 12.5, 25, 50, and 100 *μ*M FFA significantly attenuated the expression of *PPARG* (Figure 1(c)) and *CEBPA* (Figure 1(d)) at day 14, whereas FFA at 200 *μ*M did not. The inhibitory effect was attenuated with increasing FFA concentration, and 25 *μ*M FFA showed the strongest inhibitor effect on adipogenic differentiation (Figures 1(a)–1(d)). Furthermore, the presence of 1‰ DMSO did not significantly influence on the adipogenesis of hASCs *in vitro* (Supporting [Supplementary-material supplementary-material-1]).

### 3.2. FFA Inhibits Adipogenic Differentiation of hBMMSCs *In Vitro*

**Figure 2:**
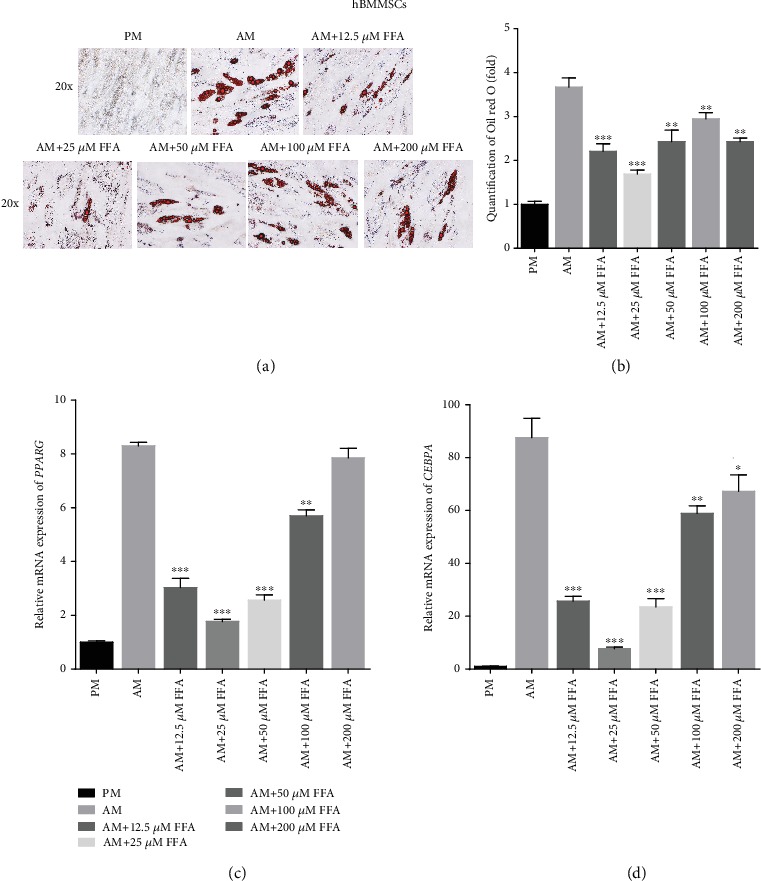
FFA inhibits adipogenic differentiation of hBMMSCs *in vitro.* (a, b) Oil red O staining and quantification showed that FFA at 12.5, 25, 50, 100, and 200 *μ*M inhibited lipid droplet formation in hBMMSCs. Human BMMSCs were treated with PM, AM, or AM+FFA at 12.5, 25, 50, 100, or 200 *μ*M for 21 days before Oil red O staining and quantification. Scale bar = 100 *μ*m. (c, d) FFA at 12.5, 25, 50, 100, and 200 *μ*M inhibited the expression of *PPARG* (c) and *CEBPA* (d) in hBMMSCs, as determined by qRT-PCR. All data are shown as the mean ± SE, *n* = 3. ^∗^*p* < 0.05, ^∗∗^*p* < 0.01, and ^∗∗∗^*p* < 0.001 compared with AM. PM: proliferation media; AM: adipogenic media; FFA: flufenamic acid; hBMMSC: human bone marrow-derived mesenchymal stem cell; *PPARG*: peroxisome proliferator activated receptor gamma; *CEBPA*: CCAAT enhancer binding protein alpha; qRT-PCR: quantitative real-time reverse transcription PCR.

To test the effects of FFA on the adipogenesis of other types of hMSCs, we treated hBMMSCs with FFA during adipogenic induction. Similar to the results obtained in hASCs, Oil red O staining and quantification showed that 12.5, 25, 50, 100, and 200 *μ*M FFA had an inhibitory effect on the adipogenic differentiation of hBMMSCs cultured in AM on day 21 (Figures 2(a) and 2(b)). Additionally, qRT-PCR showed that FFA at 12.5, 25, 50, and 100 *μ*M attenuated the expression of *PPARG* (Figure 2(c)) and *CEBPA* (Figure 2(d)) at day 14, whereas FFA at 200 *μ*M did not. The inhibitory effect was attenuated with increasing FFA concentration. Like the hASCs, treatment of hBMMSCs with 25 *μ*M FFA showed the strongest inhibitory effect on adipogenic differentiation (Figures 2(a)–2(d)). Furthermore, the presence of 1‰ DMSO did not significantly influence the adipogenesis of hBMMSCs *in vitro* (Supporting [Supplementary-material supplementary-material-1]).

### 3.3. FFA Inhibited Adipogenic Differentiation of hMSCs *In Vivo*

**Figure 3:**
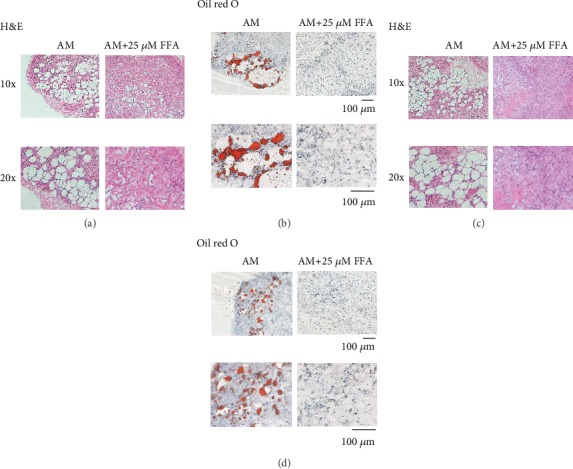
FFA inhibits adipogenic differentiation of hMSCs *in vivo.* (a, b) H&E (a) and Oil red O (b) staining of the AM and AM+FFA group of hASCs. (c, d) H&E (c) and Oil red O (d) staining of AM and AM+FFA group of hBMMSCs. Scale bar = 100 *μ*m. PM: proliferation media; AM: adipogenic media; FFA: flufenamic acid; hASC: human adipose-derived stem cell; hBMMSC: human bone marrow-derived mesenchymal stem cell; H&E.: hematoxylin and eosin.

Since 25 *μ*M FFA resulted in the optimal inhibitory effect on adipogenesis of hASCs and hBMMSCs in *in vitro*, 25 *μ*M FFA was the concentration selected for the *in vivo* experiments. Human ASCs were treated with AM or with AM+25 *μ*M FFA for 7 days, separately loaded onto Collagen Sponge scaffolds, and implanted into nude mice. Six weeks later, the implanted complexes were collected and analyzed. H&E (Figure 3(a)) and Oil red O (Figure 3(b)) staining revealed fewer lipid droplets in the FFA group. Similar results were obtained using hBMMSCs (Figures 3(c) and 3(d)).

### 3.4. FFA Suppresses Adipose Formation in OVX and Aged Mice

**Figure 4:**
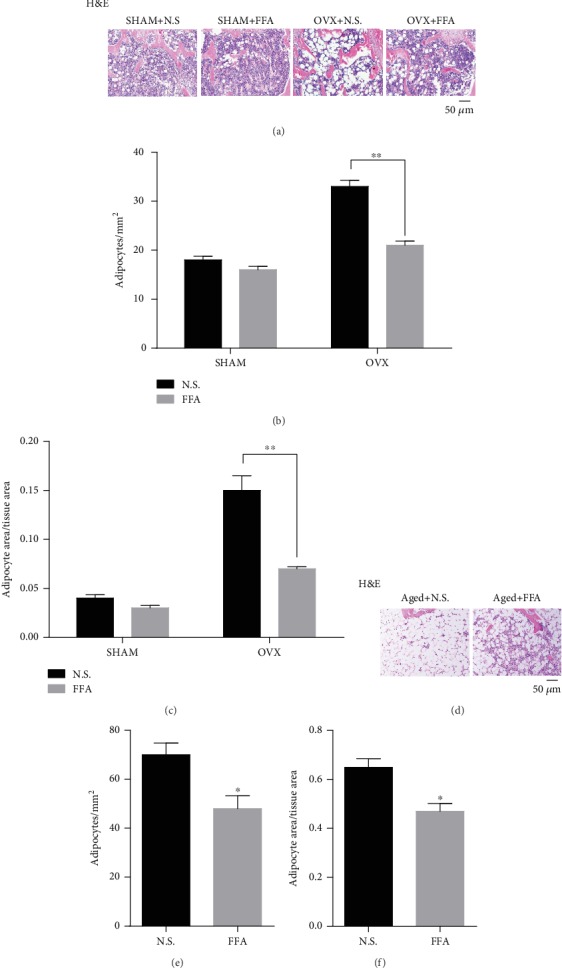
Treatment with 25 *μ*M FFA suppresses adipose formation in the bone marrow of OVX and aged mice. (a) H&E staining of femurs from SHAM mice with N.S. treatment, SHAM mice with FFA treatment, OVX mice with N.S. treatment, and OVX mice with FFA treatment for 4 weeks. (b) Adipocyte count based on H&E staining of femurs in SHAM mice with N.S. treatment, SHAM mice with FFA treatment, OVX mice with N.S. treatment, and OVX mice with FFA treatment for 4 weeks. (c) Adipocyte area/tissue area based on H&E staining of femurs in SHAM mice with N.S. treatment, SHAM mice with FFA treatment, OVX mice with N.S. treatment, and OVX mice with FFA treatment for 4 weeks. (d) H&E staining of femurs in aged mice with N.S. treatment, and aged mice with FFA treatment for 4 weeks. (e) Adipocyte count based on H&E staining of femurs in aged mice with N.S. treatment, and aged mice with FFA treatment for 4 weeks. (f) Adipocyte area/tissue area based on H&E staining images of femurs in aged mice with N.S. treatment, and aged mice with FFA treatment for 4 weeks. Scale bar = 50 *μ*m. All data are shown as the mean ± SE, *n* = 3. ^∗∗^*p* < 0.01 compared with OVX mice with N.S. treatment (b, c). ^∗^*p* < 0.05 compared with aged mice with N.S. treatment (e, f). H&E.: hematoxylin and eosin.

To further explore whether FFA could suppress adipogenesis of MSCs and adipose formation in animal disease models and define clinic value for our results, FFA was given to OVX and aged mice. Forty female mice were divided into four groups with ten mice in each group: SHAM+N.S., SHAM+FFA, OVX+N.S., and OVX+FFA. H&E staining (Figure 4(a)) showed that FFA treatment did not cause significant differences in the femurs of SHAM mice, whereas FFA treatment partially suppressed adipose formation in the bone marrow of femurs of the OVX mice. Moreover, there was a decrease in adipocyte quantity (Figure 4(b)) and adipocyte area/tissue area in the OVX mice (Figure 4(c)).

In aged mice, H&E staining indicated that FFA treatment partially attenuated adipose accumulation in the femurs (Figure 4(d)). Specifically, we observed a reduction in adipocyte quantity (Figure 4(e)), and adipocyte area/tissue area (Figure 4(f)) in the FFA group compared with the N.S group.

### 3.5. FFA Inhibits Adipogenic Differentiation of hASCs by Inhibiting the PI3K/AKT Pathway

**Figure 5:**
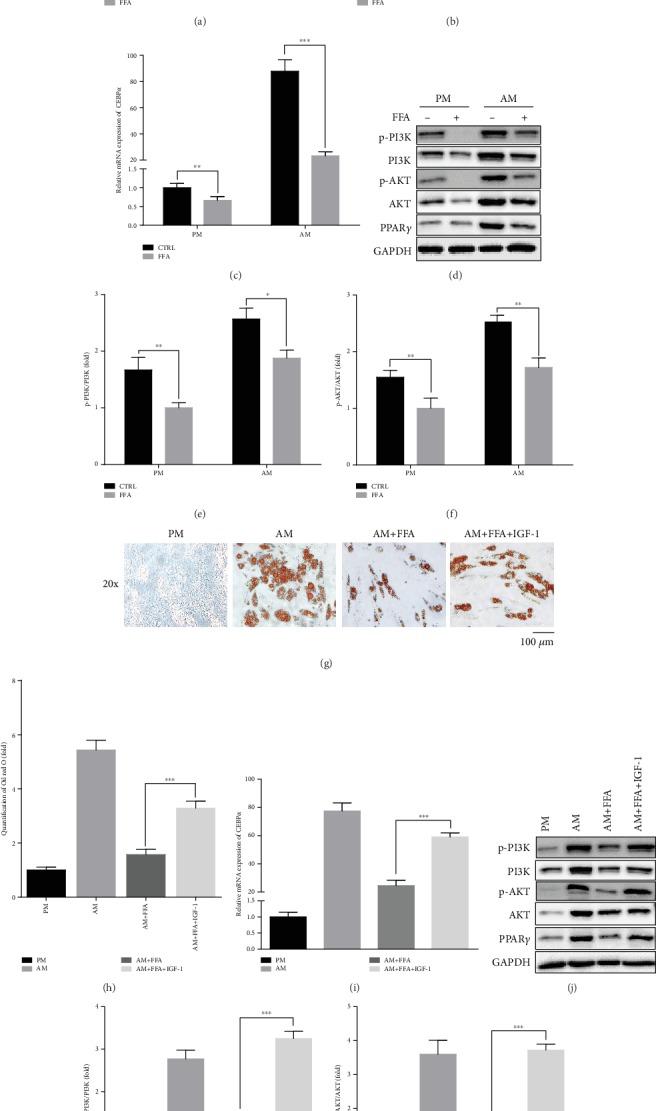
FFA inhibits adipogenic differentiation of hASCs by inhibiting AKT signaling pathway. (a–c) FFA at 25 *μ*M downregulated the expression of *FOXO1*, *FOXO3*, and *CEBPα* of hASCs grown in both PM and AM conditions, as determined by qRT-PCR. Human ASCs were treated with PM, PM+FFA at 25 *μ*M, AM, or AM+FFA at 25 *μ*M for 7 days. (d) Western blot of protein expression of p-PI3K, PI3K, p-AKT, AKT, PPAR*γ*, and the internal control, GAPDH. Human ASCs were treated with PM, PM+FFA at 25 *μ*M, AM, or AM+FFA at 25 *μ*M for 7 days. (e, f) Phosphorylated PI3K/PI3K (e) and p-AKT/AKT (f) of protein bands showed in (d). (g, h) FFA at 25 *μ*M inhibited lipid droplet formation in hASCs, but the inhibitory effect was attenuated in the presence of IGF-1(100 ng/mL), an activator of AKT signaling. Human ASCs were treated with PM, AM, AM+25 *μ*M FFA, or AM+25 *μ*M FFA, and IGF-1 for 21 days for Oil red O staining and quantification. Scale bar = 100 *μ*m. (i) Relative mRNA expression of *CEBPα*. Human ASCs were cultured in PM, AM, AM+25 *μ*M FFA, or AM+FFA at 25 *μ*M and IGF-1 for 7 days. (j) Western blot of protein expression of p-PI3K, PI3K, p-AKT, AKT, PPAR*γ*, and the internal control, GAPDH. Human ASCs were cultured in PM, AM, AM+25 *μ*M FFA, or AM+FFA at 25 *μ*M and IGF-1 for 7 days. (k, l) Phosphorylated PI3K/PI3K (k) and p-AKT/AKT (l) of proteins bands in (j). All data are presented as the mean ± SE, *n* = 3. ^∗∗^*p* < 0.01 and ^∗∗∗^*p* < 0.001 compared with CTRL (a–c, e, f). ^∗∗∗^*p* < 0.001 compared with AM+FFA (h, i, k, l). FFA: flufenamic acid; hASC: human adipose derived stem cell; PM: proliferation media; AM: adipogenic media; *FOXO1*: forkhead box protein 1; *FOXO3*: forkhead box protein 3; PI3K: phosphatidylinositol-3-kinase; *CEBPA*: CCAAT enhancer binding protein alpha; qRT-PCR: quantitative real-time reverse transcription PCR; IGF-1,: insulin-like growth factor-1.

To explore the mechanism by which FFA regulates adipogenesis, we subjected cells treated with 25 *μ*M FFA in both PM and AM to mRNA analysis for a set of key regulators of adipogenesis. Treatment with 25 *μ*M FFA suppressed the expression of the target genes of *AKT*, *FOXO1* (Figure 5(a)), *FOXO3* (Figure 5(b)), and *CEBPα* (Figure 5(c)) in hASCs cultured under PM or AM conditions. In addition, 7 days after 25 *μ*M FFA treatment, the phosphorylation of PI3K and AKT and total PI3K and AKT as well as p-PI3K/PI3K and p-AKT/AKT were downregulated in both PM and AM conditions (Figures 5(d)–5(f) and Supporting [Supplementary-material supplementary-material-1]). Additionally, FFA decreased the expression of PPAR*γ*, which was upregulated in the AM condition (Figure 5(d) and Supporting [Supplementary-material supplementary-material-1]). Based on these results, that certain concentrations of FFA could inhibit adipogenesis by inhibiting the AKT pathway. Thus, we added an activator of AKT signaling pathway, IGF-1 (100 ng/mL), to AM with 25 *μ*M FFA. Oil red O staining and quantification showed that the inhibitory effect of 25 *μ*M FFA on adipogenesis of hASCs was attenuated by IGF-1 treatment (Figures 5(g) and 5(h)). Meanwhile, qRT-PCR showed that IGF-1 upregulated the relative mRNA expression of *CEBPα* and Western blot showed that IGF-1 upregulated the phosphorylation of PI3K and AKT, total PI3K and AKT, and p-PI3K/PI3K and p-AKT/AKT as well as PPAR*γ*, which were reduced by FFA treatment alone (Figures 5(i)–5(l) and Supporting [Supplementary-material supplementary-material-1]).

## 4. Discussion

In the present study, we found that treatment with FFA inhibited adipogenesis of two kinds of hMSCs both *in vitro* and *in vivo*. The inhibitory effect was attenuated as the concentration of FFA increased, and 25 *μ*M FFA showed the optimal inhibitory effect on adipogenesis. Mechanistically, FFA inhibited adipogenesis of hMSCs by inhibiting the AKT pathway. These data indicate the potential application of FFA in tissue engineering and diseases related to excessive adipogenic differentiation of MSCs.

The *in vitro* experiments of this study show that FFA inhibits adipogenesis of hMSCs. According to the qRT-PCR results, the inhibitory effects of FFA on the expression of adipogenesis markers declined as the concentration of FFA increased from 25 *μ*M to 200 *μ*M. However, Oil red O staining showed that 200 *μ*M FFA inhibited adipogenesis better than 100 *μ*M FFA. This may be because 200 *μ*M FFA also inhibited the proliferation of hMSCs, which has been documented in our previous study [25]. Human ASCs and hBMMSCs displayed similar results both *in vitro* and *in vivo*. This might be because they are both populations of postnatal tissue-specific stem cells with similar gene expression profiles and display similar characteristics [28]. We found that low concentrations of FFA promote osteogenesis of hMSCs [25], and adipogenic differentiation is generally inhibited when osteogenic differentiation is promoted. The results of our present study are in agreement with our previous report. These data indicated that FFA treatment could play a role in mediating the lineage differentiation of hMSCs. In support of these *in vitro* results, we showed that FFA suppressed adipogenesis of hMSCs *in vivo*. This evidence indicates that FFA could be a potential therapeutic option to treat diseases related to excessive adipogenic differentiation of MSCs.

Excessive adipogenic differentiation of MSCs can be seen in several diseases, such as obesity and osteoporosis [9, 29, 30]. Studies have shown that aging and obesity promote the adipogenesis of MSCs, increase accumulation of marrow adipose tissue (MAT), and impair the osteogenic functions of MSCs [31, 32]. As the only tissue where bone and fat coexist in the same microenvironment, bone marrow is an excellent window to study stem cell lineage commitment. We observed that treatment of low concentrations of FFA suppressed MAT accumulation in OVX and aged mice. Loss of bone and increase of adipose tissue in the bone marrow are signs of bone aging. Therefore, these data demonstrate that FFA treatment may mediate the adipogenesis of hMSCs and could be useful to delay bone aging.

NSAIDs are often used to treat osteoarthritis and other bone-related diseases, and some studies have reported that NSAID drugs effect the biological behavior of bone and MSCs, including adhesion, proliferation, and differentiation. However, few studies have reported on the effects of NSAIDs on the adipogenesis of MSCs except that indomethacin promotes adipogenesis of MSCs [16]. Furthermore, little is known about the effects of the fenamic acids on the differentiation of MSCs. FFA is a representative of the fenamic acids and has been used historically to treat a set of common diseases in the clinic [33, 34]. FFA is easily obtained and is inexpensive. The present study provides insights into the potential application of FFA as a new, safe, and economical option in regulating adipogenic differentiation of MSCs and in treatment of diseases related to excessive adipogenic differentiation of MSCs.

Our study indicated that FFA inhibited adipogenesis of hMSCs by inhibiting the PI3K/AKT pathway. The AKT pathway is closely associated with the lineage commitment of MSCs [19, 21]. Previous studies have shown that AKT signaling is upregulated during adipogenic differentiation [19]. Our findings are in agreement with previous reports, and we are the first to point out that FFA inhibits adipogenic differentiation through the PI3K/AKT pathway. Although the mechanism of how FFA inhibits adipogenic differentiation of hMSCs has not been completely clarified, it is mediated, at least partly, by the inhibiting PI3K/AKT pathway.

Nevertheless, there are several limitations of our study. This study only examined the signaling pathways by which certain concentrations FFA inhibit adipogenic differentiation of hMSCs. Why the inhibitory effect declines as FFA concentration increases remains unclear. This might be because low concentrations of FFA and high concentrations of FFA influenced adipogenesis of hMSCs by different mechanism and we will conduct deeper exploration in future study. Furthermore, to promote the clinical transformation of our findings, further exploration is needed to determine the effective concentration for treatment in humans.

Overall, our results indicate that FFA can inhibit the adipogenesis of hMSCs *in vitro* and *in vivo* by inhibiting the PI3K/AKT pathway. These data suggest that FFA is applicable in regulating adipogenesis of MSCs and highlight FFA as a potential therapeutic option to treat diseases related to excessive adipogenic differentiation of MSCs.

## 5. Conclusion

FFA inhibits adipogenesis of hMSCs *in vitro*, at an optimal inhibitory concentration of 25 *μ*M FFA. Treatment with 25 *μ*M FFA also inhibits adipogenesis of hMSCs *in vivo*. Mechanistically, FFA inhibits adipogenesis through inhibition of the PI3K/AKT signaling pathway. These data suggest that FFA might be used to inhibit adipogenesis of human MSCs in tissue engineering and diseases related to excessive adipogenic differentiation of MSCs.

## Figures and Tables

**Table 1:** List of primers used in this study.

Gene	Forward primer (5′-3′)	Reverse primer (5′-3′)
*GAPDH*	GAAGGTGAAGGTCGGAGTC	GAAGATGGTGATGGGATTTC
*FOXO1*	AAGAGCGTGCCCTACTTCAA	TTCCTTCATTCTGCACACGA
*FOXO3*	GGTGCGTTGCGTGCCCTACT	CCGTGGCAGTTCCACCGTGC
*PPARγ*	GAGGAGCCTAAGGTAAGGAG	GTCATTTCGTTAAAGGCTGA
*C/EBPα*	GGGCCAGGTCACATTTGTAAA	AGTAAGTCACCCCCTTAGGGTAAGA

## Data Availability

The data used to support the findings of this study are available from the corresponding author upon request.

## Supplementary Materials

Supplementary Materials Supporting Figure 1: 1‰ DMSO did not affect lipid droplet formation in hMSCs. Supporting Figure 2: quantitative analysis of proteins bands in Figure 5 ARRIVE checklist.

## References

[1] Koch T. G., Berg L. C., Betts D. H. (2009). Current and future regenerative medicine - principles, concepts, and therapeutic use of stem cell therapy and tissue engineering in equine medicine. *The Canadian Veterinary Journal*.

[2] Pittenger M. F., Mackay A. M., Beck S. C. (1999). Multilineage potential of adult human mesenchymal stem cells. *Science*.

[3] Jiang Y., Jahagirdar B. N., Reinhardt R. L. (2002). Pluripotency of mesenchymal stem cells derived from adult marrow. *Nature*.

[4] Leo A. J., Grande D. A. (2006). Mesenchymal stem cells in tissue engineering. *Cells, Tissues, Organs*.

[5] Barzelay A., Weisthal Algor S., Niztan A. (2018). Adipose-derived Mesenchymal stem cells migrate and rescue RPE in the setting of oxidative stress. *Stem Cells International*.

[6] Yang X., Li Y., Liu X., Zhang R., Feng Q. (2018). In vitro uptake of hydroxyapatite nanoparticles and their effect on osteogenic differentiation of human mesenchymal stem cells. *Stem Cells International*.

[7] Hotta K., Matsuzawa Y. (2001). Molecular mechanism in the development of the complications associated with obesity--the physiological and pathological role of adipocytokines. *Nihon Rinsho*.

[8] Farooqi S. I. (2015). Genetic, molecular and physiological mechanisms involved in human obesity: Society for Endocrinology Medal Lecture 2012. *Clinical Endocrinology*.

[9] Zhao L. J., Jiang H., Papasian C. J. (2008). Correlation of obesity and osteoporosis: effect of fat mass on the determination of osteoporosis. *Journal of Bone and Mineral Research*.

[10] Moncada S., Ferreira S. H., Vane J. R. (1973). Prostaglandins, aspirin-like drugs and the oedema of inflammation. *Nature*.

[11] Vane J. R. (1971). Inhibition of prostaglandin synthesis as a mechanism of action for aspirin-like drugs. *Nature: New Biology*.

[12] Vane J. R. (1976). The mode of action of aspirin and similar compounds. *The Journal of Allergy and Clinical Immunology*.

[13] Sriram D., Yogeeswari P. (2010). *Medicinal Chemistry*.

[14] Liu H., Li W., Liu Y., Zhang X., Zhou Y. (2015). Co-administration of aspirin and allogeneic adipose-derived stromal cells attenuates bone loss in ovariectomized rats through the anti-inflammatory and chemotactic abilities of aspirin. *Stem Cell Research & Therapy*.

[15] Hadjicharalambous C., Alexaki V. I., Alpantaki K., Chatzinikolaidou M. (2016). Effects of NSAIDs on the osteogenic differentiation of human adipose tissue-derived stromal cells. *The Journal of Pharmacy and Pharmacology*.

[16] Styner M., Sen B., Xie Z., Case N., Rubin J. (2010). Indomethacin promotes adipogenesis of mesenchymal stem cells through a cyclooxygenase independent mechanism. *Journal of Cellular Biochemistry*.

[17] Berndt N., Yang H., Trinczek B. (2010). The Akt activation inhibitor TCN-P inhibits Akt phosphorylation by binding to the PH domain of Akt and blocking its recruitment to the plasma membrane. *Cell Death and Differentiation*.

[18] Davis W. J., Lehmann P. Z., Li W. (2015). Nuclear PI3K signaling in cell growth and tumorigenesis. *Frontiers in Cell and Development Biology*.

[19] Nakae J., Kitamura T., Kitamura Y., Biggs W. H., Arden K. C., Accili D. (2003). The forkhead transcription factor Foxo1 regulates adipocyte differentiation. *Developmental Cell*.

[20] Jing E., Gesta S., Kahn C. R. (2007). SIRT2 regulates adipocyte differentiation through Fox O1 acetylation/deacetylation. *Cell Metabolism*.

[21] Cypess A. M., Zhang H., Schulz T. J. (2011). Insulin/IGF-I regulation of necdin and brown adipocyte differentiation via CREB- and Fox O1-associated pathways. *Endocrinology*.

[22] Peng X. D., Xu P. Z., Chen M. L. (2003). Dwarfism, impaired skin development, skeletal muscle atrophy, delayed bone development, and impeded adipogenesis in mice lacking Akt 1 and Akt 2. *Genes & Development*.

[23] Ducy P., Desbois C., Boyce B. (1996). Increased bone formation in osteocalcin-deficient mice. *Nature*.

[24] Jin C., Zhang P., Zhang M. (2017). Inhibition of SLC7A11 by sulfasalazine enhances osteogenic differentiation of mesenchymal stem cells by modulating BMP2/4 expression and suppresses bone loss in ovariectomized mice. *Journal of Bone and Mineral Research*.

[25] Liu X., Li Z., Liu H. (2019). Low concentration flufenamic acid enhances osteogenic differentiation of mesenchymal stem cells and suppresses bone loss by inhibition of the NF-*κ*B signaling pathway. *Stem Cell Research & Therapy*.

[26] Dempster D. W., Compston J. E., Drezner M. K. (2013). Standardized nomenclature, symbols, and units for bone histomorphometry: a 2012 update of the report of the ASBMR Histomorphometry Nomenclature Committee. *Journal of Bone and Mineral Research*.

[27] Yu B., Huo L., Liu Y. (2018). PGC-1*α* controls skeletal stem cell fate and bone-fat balance in osteoporosis and skeletal aging by inducing TAZ. *Cell Stem Cell*.

[28] Lee R. H., Kim B., Choi I. (2004). Characterization and expression analysis of mesenchymal stem cells from human bone marrow and adipose tissue. *Cellular Physiology and Biochemistry*.

[29] Rosen C. J., Bouxsein M. L. (2006). Mechanisms of disease: is osteoporosis the obesity of bone?. *Nature Clinical Practice Rheumatology*.

[30] Zhao L. J., Liu Y. J., Liu P. Y., Hamilton J., Recker R. R., Deng H. W. (2007). Relationship of obesity with osteoporosis. *The Journal of Clinical Endocrinology and Metabolism*.

[31] Ambrosi T. H., Scialdone A., Graja A. (2017). Adipocyte accumulation in the bone marrow during obesity and aging impairs stem cell-based hematopoietic and bone regeneration. *Cell Stem Cell*.

[32] Fan Y., Hanai J. I., le P. T. (2017). Parathyroid hormone directs bone marrow mesenchymal cell fate. *Cell Metabolism*.

[33] Barnardo D. E., Currey H. L., Mason R. M., Fox W. R., Weatherall M. (1966). Mefenamic acid and flufenamic acid compared with aspirin and phenylbutazone in rheumatoid arthritis. *British Medical Journal*.

[34] Rajan K. T., Hill A. G., Barr A., Whitwell E. (1967). Flufenamic acid in rheumatoid arthritis. *Annals of the Rheumatic Diseases*.

