# Supplementary material for: Flufenamic Acid Inhibits Adipogenic Differentiation of Mesenchymal Stem Cells by Antagonizing the PI3K/AKT Signaling Pathway
Source: Stem Cells Int. 2020 Mar 16;2020:1540905. doi: 10.1155/2020/1540905 (PMC7102471; doi:10.1155/2020/1540905)
Supplement: Supplementary Materials — Supporting Figure 1: 1‰ DMSO did not affect lipid droplet formation in hMSCs. Supporting Figure 2: quantitative analysis of proteins bands in Figure 5 ARRIVE checklist. [file 1540905.f1.zip › Supporting Figures.docx]

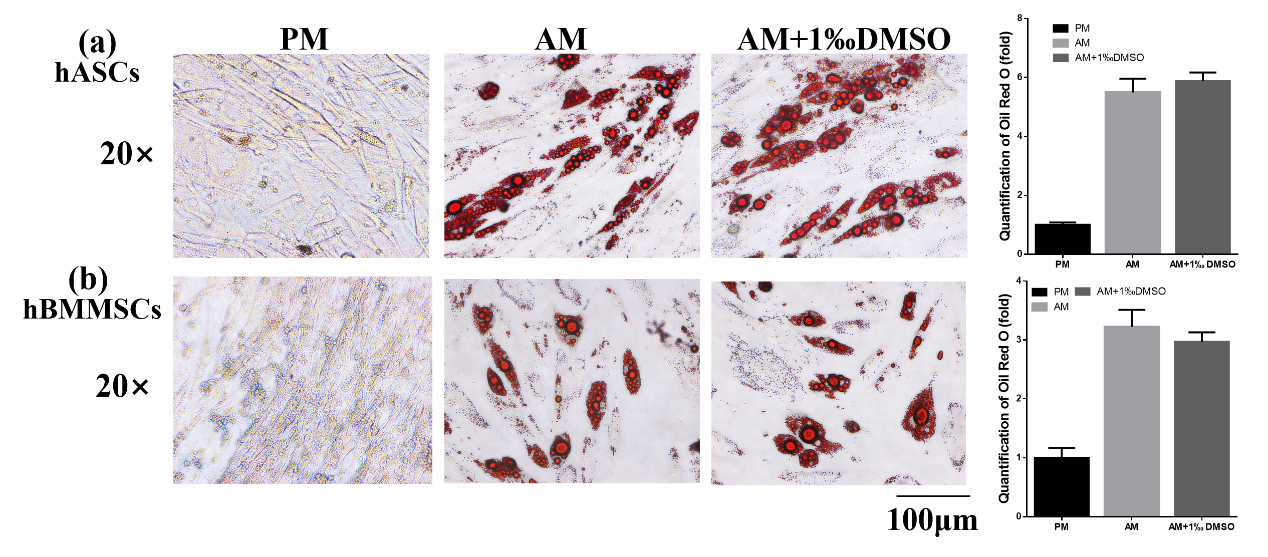


Supporting Figure 1. 1‰ DMSO did not affect lipid droplet formation in hMSCs (a, b) 1‰ DMSO did not affect the lipid droplet formation in hASCs (a) or hBMMSCs (b). Scale bar = 100 μm. All data are presented as the mean ± SE, n = 3. FFA, flufenamic acid; hASC, human adipose-derived stem cell; hBMMSC, human bone marrow-derived mesenchymal stem cell; PM, proliferation media; AM, adipogenic media.


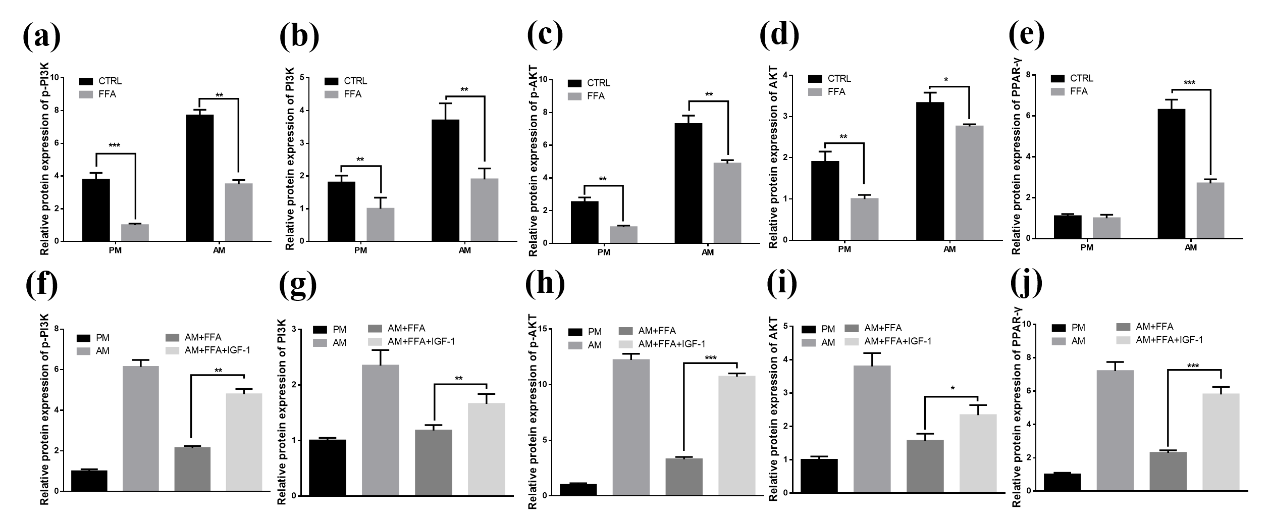


Supporting Figure 2. (a-e) Quantitative analysis of proteins bands in Figure 5(d). (f-j) Quantitative analysis of proteins bands in Figure 5(j) All data are presented as the mean ± SE, n = 3, **p* < 0.05, ***p* < 0.01, and ****p* < 0.001. FFA, flufenamic acid; PM, proliferation media; AM, adipogenic media.
